# The effectiveness of split tablet dosing versus alternate-day dosing of warfarin: a randomized control trial

**DOI:** 10.1038/s41598-021-03606-z

**Published:** 2021-12-15

**Authors:** Chanyapat Kaewsaengeak, Usanee Pienputtarapong, Teerapong Tocharoenchok

**Affiliations:** 1 Department of Surgery, Police General Hospital, Bangkok, 10330 Thailand; 2 Ambulatory Care Unit, Pharmacy Department, Faculty of Medicine Siriraj Hospital, Mahidol University, Bangkok, 10700 Thailand; 3 Division of Cardiothoracic Surgery, Department of Surgery, Faculty of Medicine Siriraj Hospital, Mahidol University, Bangkok, 10700 Thailand

**Keywords:** Randomized controlled trials, Clinical pharmacology

## Abstract

Due to large dosage variation, a variety of warfarin prescription regimens are utilized for specific doses such as tablet splitting, or pill strength alternating. The clinical comparison between the two is lacking. We hypothesize that both approaches result in different times in therapeutic range. We randomized patients with specific warfarin dosage and stable INR for 6 months or longer to receive the whole tablet, alternate-day dosing or the split tablet, same daily-dosing regimen without initial dose change and followed them every 6 weeks for 6 months. The primary outcome was a time in therapeutic range of 2.0–3.0. The secondary outcomes included dosage, compliance, INR, anticoagulant-related events. A total of 66 patients were enrolled, 32 randomly assigned to the split tablet regimen (group S) and 34 to the alternate-day regimen (group A) with two withdrawers. The mean age was 58.6 ± 8.5 years. All baseline characteristics of both groups were similar. The average time in therapeutic range was 72.8 ± 25.4% in group S and 74.9 ± 22.0% in group A (*p* = 0.72). There were no significant differences in warfarin dosage, compliance, INR and, complications between the two groups. Both warfarin prescription methods, the split tablet and the alternate-day had comparable time in the therapeutic range.

## Introduction

Warfarin is one of the most used oral anticoagulants despite its narrow therapeutic index and wide variation in the maintenance dose among patients. Even though non-vitamin k antagonist oral anticoagulants (NOAC) are now widely available, a significant portion of patients still requires this vitamin k antagonist (VKA) especially for their mechanical valve protheses or valvular atrial fibrillation^1^. According to the pharmacological mechanism, warfarin anticoagulant activity expresses via interfering vitamin k-dependent clotting factors synthesis. Due to this indirect action and the delay in exerting its full efficacy, maintenance dose adjustment is recommended based on a weekly dose basis^2^. Although there are several tablet strengths, to achieve a particular weekly dose, numbers of ‘less than ideal’ prescription regimens are frequently required, such as tablet splitting, irregular dosing schedule, or a fancy combination of both. Up to the present, there is a paucity of evidence from clinical studies regarding their effectiveness. Some in vivo studies reported weight inaccuracy of tablet splitting while inconsistent dose schedule could jeopardize drug compliance or may cause dosing error^3,4^. We hypothesize that the alternate-day dosing provides better international normalized ratio (INR) control than the split tablet approach, so a randomized controlled trial is conducted to evaluate the effect of two prescription methods on the time in therapeutic range (TTR).

## Methods

### Trial design

The study is a parallel-grouped, active comparator, randomized controlled trial, approved by Siriraj Institutional Review Board on 25/07/2017, which is in full compliance with international guidelines for human research protection such as the Declaration of Helsinki (Study Code 320/2560(EC2)). The study was registered in the Thai Clinical Trials Registry on 6/12/2016 and posted on 12/12/2016 as TCTR20161212001. All participants provided written informed consent before the study and were compensated for their time and participation. All methods were performed in accordance with the relevant guidelines and regulations.

### Participants

The anticoagulated patients who were followed up at the outpatient clinic of the Division of Cardio-Thoracic Surgery, Department of Surgery, Faculty of Medicine Siriraj Hospital, Mahidol University, Thailand from January 2018 to December 2020 were enrolled. Eligible patients had six months or longer history of stable therapeutic INR levels of 2.0–3.0 achieved by the specific weekly dose of warfarin in the dosage protocol (Table 1) and could comply with 6 months follow up protocol. The patients whose a period of warfarin discontinuation was possibly needed were excluded.

**Table 1:** Warfarin dosing protocol.

Doses (mg/week)	Group S prescription	Group A prescription
14	3 mg ¼ tab po hs and 5 mg ¼ tab po hs	2 mg 1 tab po hs Mo, We, Th, Sa 3 mg 1 tab po hs Tu, Fr
17.5	5 mg ½ tab po hs	2 mg 1 tab po hs ad (odd date) 3 mg 1 tab po hs ad (even date)
21	2 mg 1½ tab po hs	2 mg 1 tab po hs ad (odd date) 2 mg 2 tab po hs ad (even date)
24.5	2 mg ½ tab po hs and 5 mg ½ tab po hs	3 mg 1 tab po hs ad (odd date) 2 mg 2 tab po hs ad (even date)
28	3 mg ½ tab po hs and 5 mg ½ tab po hs	3 mg 1 tab po hs ad (odd date) 5 mg 1 tab po hs ad (even date)
31.5	3 mg 1½ tab po hs	5 mg 1 tab po hs ad (odd date) 2 mg 2 tab po hs ad (even date)

*Fr* Friday, *Mo* Monday, *Sa* Saturday, *Th* Thursday, *Tu* Tuesday, *We* Wednesday.

### Randomization

This was a 2-armed parallel trial with a 1:1 allocation ratio to receive either the split tablet, same daily-dosing regimen (Group S) and to receive the whole tablet, alternate-day dosing regimen (Group A). By the order of enrollment, after informed consent, a member of the pharmacists of our research team assigned the patients to the group according to the randomization codes that were computer-generated from Siriraj Routine to Research Unit in advance through simple random sampling. Neither patients nor investigators were blinded to the group assignment except for the INR outcome assessors.

### Procedure

The weekly dosage of warfarin for all participants remains unchanged while the prescription method would be adjusted according to the study protocol (Table 1), depending on the patient’s group. The pill cutter was provided in group S and the dosing calendar was applied to the patients in group A to minimize dosing errors. All participants were asked to follow up on their INR level and anticoagulant-related adverse event at the 6-week interval for a total of 6 months. If the INR was out of the therapeutic level without the explainable factors, such as drug-interaction, food-interaction, or compliance; the dosage adjustment would be commenced per-protocol (Supplementary Table S1) with respect to the randomized prescription methods. All participants were instructed to strictly follow the regimen and the follow-up protocol. After the fourth INR measurement, the time in therapeutic range (TTR) was then calculated.

### Sample size

Based on the pre-study survey in 102 selected patients at our warfarin clinic, 65.7 ± 21.1% and 81.4 ± 22.2% TTR were expected in the S and A groups, respectively. A sample size of 30 in each group was estimated to have 80% power, with a significance level of 0.05 for a 2-sided test of the mean difference between 2 independent groups. To compensate for 10% dropouts, the target enrolment was set at 33 patients per group, for a total of 66 patients.

### Outcome measures

The primary study outcome, TTR, was calculated according to the Rosendaal method^5^. In short, the frequency of the INR measurements and the actual levels are incorporated assuming that the changes between the measurements are linear over time. The TTR reflexes the quality of INR maintenance^6,7^. The secondary outcomes were the dosage change incidence, INR levels, the bleeding and thromboembolic events of the warfarin, the compliance and adherence of the participants to the study protocol. Poor compliance was defined in the presence of missed dose, wrong dose, or recall uncertainty.

### Statistical analysis

The summary statistics of the patient baseline characteristics were calculated by the patient group. The continuous variables were reported in mean and standard deviation, the qualitative data were analyzed and reported in count and percentage. The variables between the two study groups were compared using unpaired t-tests or chi-squared tests (or the non‐parametric equivalents where appropriate), with statistical significance defined as *p* < 0.05. The primary analyses were performed in standard the intention-to-treat population, which included all patients who were randomly assigned to a study group. Missing TTR data of the withdrawers were imputed according to the baseline-value-carried-forward method using data from a pre-study pilot survey. Sensitivity analyses were also performed for the primary outcome using an extreme case scenario. All analyses were performed with the use of SPSS™ software version 20.0 (SPSS Inc., IBM Company, Chicago, Illinois, USA).

## Results

**Figure 1:**
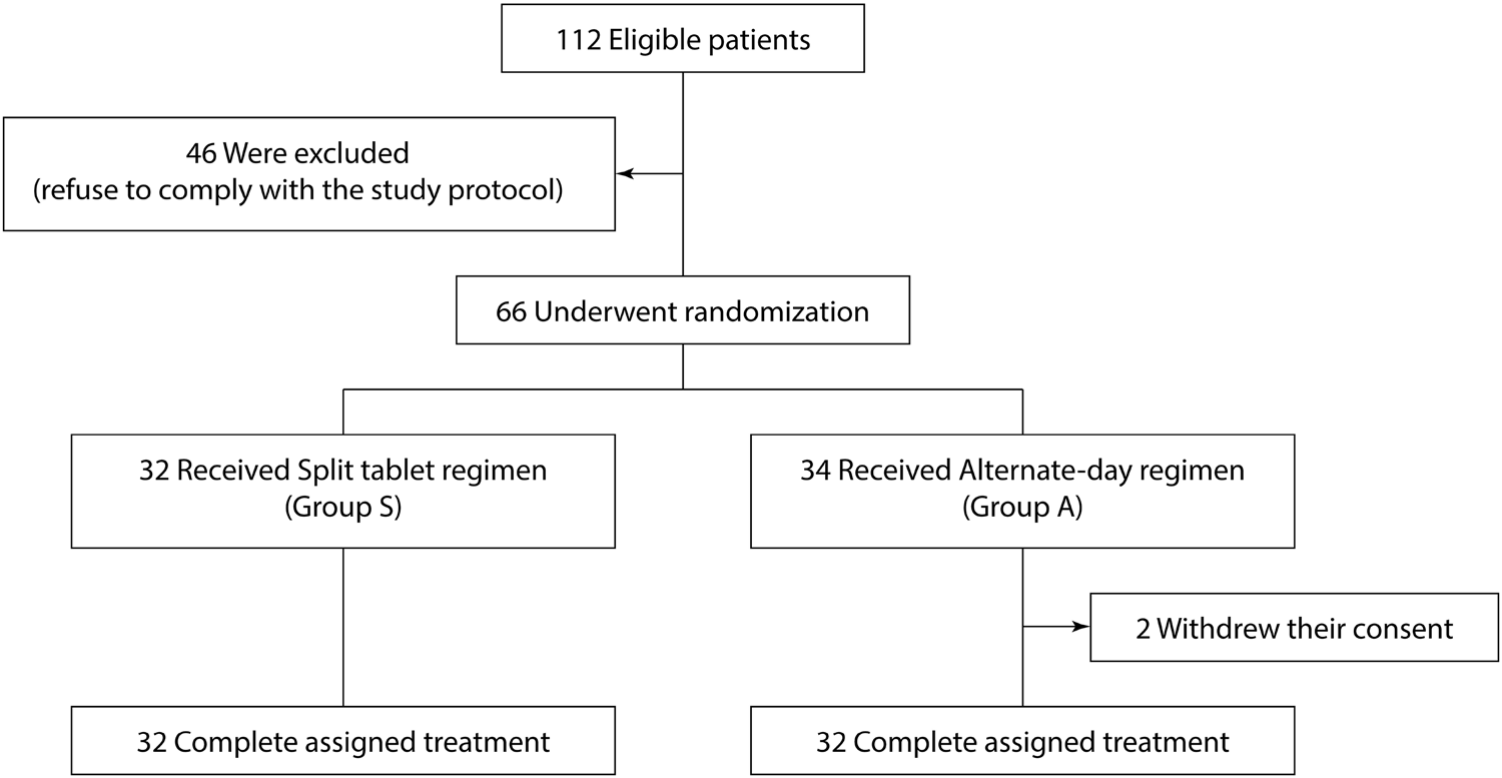
Enrollment, randomization, and assigned treatment.

A total of 112 eligible patients were informed of the study enrollment during the study period. After the exclusion of 46 patients due to reluctance to comply with the study protocol, the rest 66 patients were randomized into two groups. Thirty-two participants were assigned to group S, while 34 participants were randomized to group A with two of them later refusing to comply with the follow-up protocol and withdrawing their consent. All the rest participants were able to adhere to the study protocol without crossover or drop-off (Fig. 1). Table 2 shows baseline characteristics of patients included in the intention-to-treat analysis; there were no significant differences among the study groups. The mean age of the entire cohort was 58.6 ± 8.5 years with 47.0% of the male gender. Mechanical valve thrombosis prevention was by far the most common indication to anticoagulate at 95.5% of the study population.

**Table 2:** Baseline characteristics in the intention-to-treat population.

Parameters	Group S (n = 32)	Group A (n = 34)	p-value
Age, years	58.0 ± 7.3	59.1 ± 9.7	0.61
Male gender, n (%)	14 (43.8)	17 (50.0)	0.61
**Underlying disease, n (%)**			
Atrial fibrillation	11 (34.4)	13 (38.2)	0.80
Hypertension	10 (31.2)	11 (32.4)	1.00
Dyslipidemia	8 (25.0)	5 (14.7)	0.36
Diabetes mellitus	3 (9.4)	3 (8.8)	1.00
Old cerebrovascular accident	2 (6.2)	3 (8.8)	1.00
Coronary atherosclerosis	0 (0)	4 (11.8)	0.11
**Primary indication for warfarin treatment, n (%)**			0.49
Prosthetic valve	31 (96.9)	32 (94.1)	
Deep vein thrombosis	0 (0.0)	2 (5.9)	
Atrial fibrillation	1 (3.1)	0 (0)	
Smoking, n (%)	2 (6.2)	0 (0)	0.23
Alcohol consumption, n (%)	4 (12.5)	0 (0)	0.05
Warfarin dose, mg/week	23.3 ± 5.4	21.2 ± 5.4	0.12
INR before randomization	2.50 ± 0.25	2.44 ± 0.30	0.40
Duration of INR 2–3 without dose change, months	10.3 ± 3.9	13.0 ± 7.7	0.08
Caregiver dependent, n (%)	0 (0)	4 (11.8)	0.11

Plus-minus values are means ± standard deviation.

### Primary outcome

**Figure 2:**
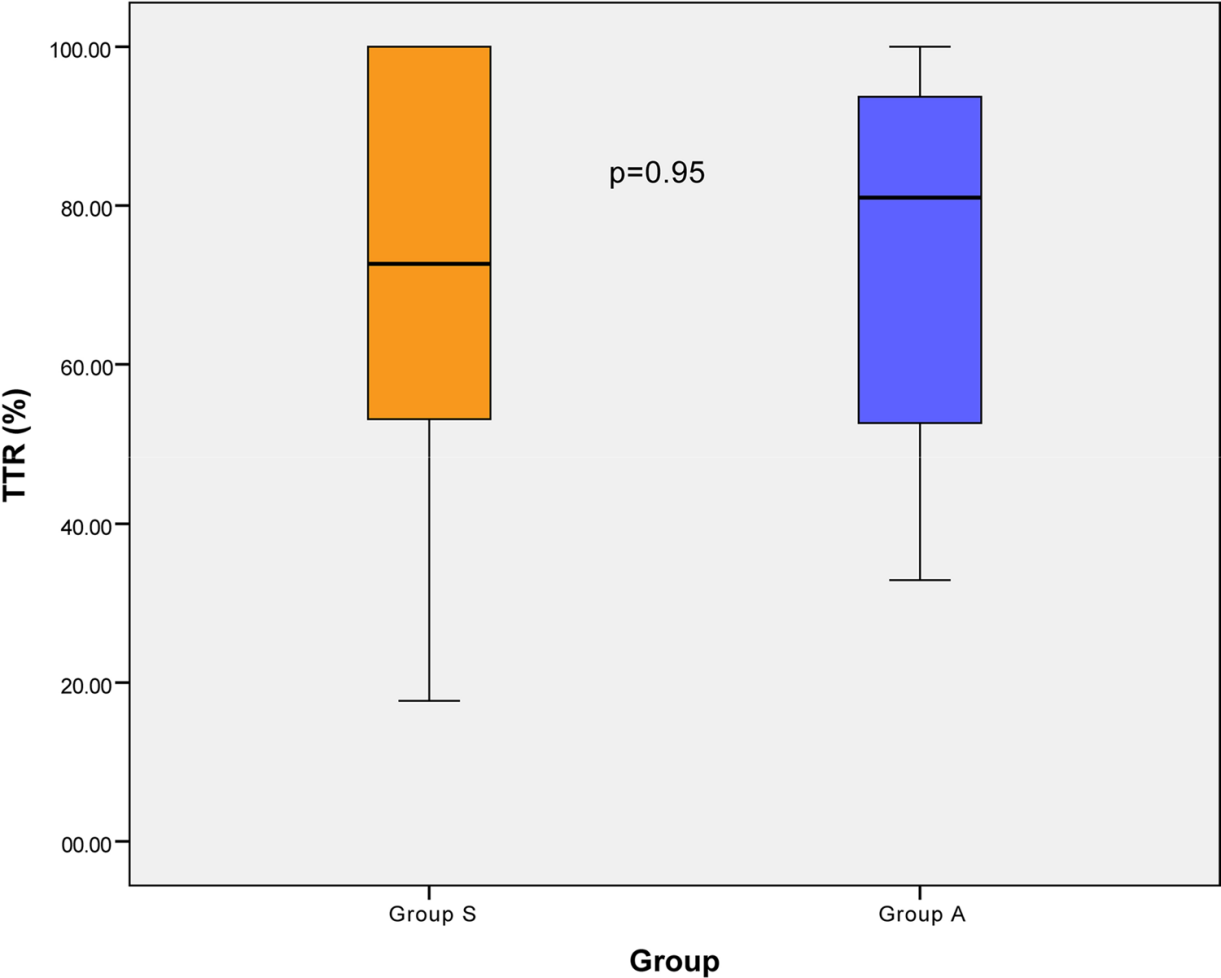
Box-and-Whisker plot of the time in therapeutic range.

The overall average TTR was 73.9 ± 23.5% in the intention-to-treat population with a difference of − 2.1 percentage points between the groups (95% confident interval − 13.7 to 9.6) (Table 3 and Fig. 2).

**Table 3:** Study outcomes in the intention-to-treat and as-treated population.

Parameters	Group S	Group A	p-value
**Primary outcome (intention-to-treat population)**	(n = 32)	(n = 34)	
Mean time in therapeutic range, %	72.8 ± 25.4	74.9 ± 22.0	0.72
**Secondary outcomes (as-treated population)**	(n = 32)	(n = 32)	
Dose adjustment for inadequate INR, n (%)	1 (3.1)	6 (18.8)	0.10
Dose adjustment for excessive INR, n (%)	5 (15.6)	5 (15.6)	1.00
Average INR during the study period	2.45 ± 0.37	2.53 ± 0.36	0.39
INR change from the baseline value	0.05 ± 0.43	− 0.09 ± 0.41	0.20
Minor bleeding event, n (%)	6 (18.8)	10 (31.2)	0.25
Major bleeding event, n (%)	0 (0)	0 (0)	n/a
Thromboembolic event, n (%)	0 (0)	0 (0)	n/a
Participants with poor compliance, n (%)	3 (9.4)	3 (9.4)	1.00
Participants with drug or food interaction, n (%)	6 (18.8)	7 (21.9)	0.76

Plus-minus values are means ± standard deviation.

IQR interquartile range.

Despite a small proportion of post-randomization withdrawal (2 of 66; 3%) in group A, a sensitivity analysis has been performed to affirm the validity of the primary outcome. The calculated TTR for extreme values in the alternative-day regimen were 76.0 ± 22.7 and 70.1 ± 28.2 for the best and worst possible missing TTR value, respectively. Those still rendered no statistical difference in TTR between the two groups (*p* = 0.59, *p* = 0.68; respectively).

### Secondary outcomes

Most of the patients could maintain the INR level without dose adjustment, however, 16 patients needed the dose adjustment (5 in group S and 11 in group A; *p* = 0.11). Despite reported minor bleedings (such as ecchymosis and bleeding per gum) in one-fourth of the participants, there was no reported major bleeding or thromboembolic event. Six patients (9.4%), three from each study arm, could not fully comply with the treatment protocol. One-fifth of the patients experienced food or drug interaction during the study period (Table 3).

## Discussion

We found no difference in the time in therapeutic range between the tablet splitting group and the alternate-day group. Based on this finding, one could speculate that when the patient needs a particular dose of warfarin without available tablet strength, pill cutting using a tablet splitter is an equivalent alternative to the irregular dosing regimen with aid of a dosing calendar. To our knowledge, this is the first prospective In Vivo comparison between the two warfarin prescription methods.

The reported TTR in the patient with warfarin treatment varies from 30 to 70%^7,8^ with the desired value of 60–70% or above^9,10^. This indicates good control of INR in the majority of patients who follow the prescription protocol at our pharmacist-managed anticoagulation clinic regardless of the prescription method.

Despite discouraging results of pill splitting studies with dosage deviation of 9–37%^11–13^, if the inaccuracy attributes primarily from the weight variation in split tablets (not from tablets powder or fragment loss during the splitting process), this effect would be diluted day-by-day as warfarin has protracted blood-thinning effect. This idea supports the need for tablet scoring and the pill-splitter.

Concerning alternative-day dosing or other complex regimens to avoid tablet split, one study found that this approach was associated with 7% dosing confusion and 14% dosing error^14^ while another study found fewer patients compliant with the split tablet regimen but no relationship to the alternating dosages^15^. The latter study also found that combining tablet split with alternating dose did worse in terms of regimen satisfaction and possible subsequent poorer INR control^15^. This idea supports the use of a dosing calendar.

A significant portion of the eligible patients (41%) were excluded mainly because they refused to comply with the closed follow-up schedule of 6-week intervals (as opposed to several months as all of them had good control of their INR—indicative of good warfarin compliance). All exclusions took place before the randomization process which should balance any selection bias.

It seems to be impractical to double-blind the current study. However, because the dosing and follow-up were based on a pre-defined protocol, we believe that the care of patients was not influenced by the assigned treatment. In addition, as we were using an objective endpoint (i.e. INR), we do not believe that the primary outcome was biased.

This result was obtained from a strict control environment in a selected patient subgroup, however, to apply it to the general care setting without a tablet cutter, dosing calendar and regular pharmacists’ advisories, great care have to be exercised. Also, our study might be subjected to underpower from the small sample size. A larger study might be needed to confirm our results.

## Conclusion

In summary, the alternate-day dosing and the split tablet approach had comparable time in the therapeutic ranges. Our study supports either prescription methods (but not the combination) when a specific warfarin dose could not be obtained by the accessible tablet strength whichever the patient satisfies.

## Supplementary Information

Supplementary Table S1.

## Data Availability

The datasets generated during and/or analysed during the current study are available from the corresponding author on reasonable request.
