# Supplementary material for: The effectiveness of split tablet dosing versus alternate-day dosing of warfarin: a randomized control trial
Source: Sci Rep. 2021 Dec 15;11:24060. doi: 10.1038/s41598-021-03606-z (PMC8674310; doi:10.1038/s41598-021-03606-z)
Supplement: Supplementary file 1 — Supplementary Table S1. [file 41598_2021_3606_MOESM1_ESM.docx]

| Initial dose  (mg/week) | Group | INR 4-4.9 | | INR 3.1-3.9 | | INR 1.5-1.9 | | INR <1.5 |
| --- | --- | --- | --- | --- | --- | --- | --- | --- |
| 14 | **S**  **A** | (5) ¼ + (2) ¼  2/3/-/2/3/2/- | (5) ¼ + (2) ¼  2/3/-/2/3/2/- | | (5) ¾  2/3/2/3/2/3/- | | (5) ¾  2/3/2/3/2/3/2 | |
| 17.5 | **S**  **A** | (5) ¾  2/3/2/3/2/3/- | (5) ¾  2/3/2/3/2/3/2 | | (5) ¼ + (3) ½  3/2/3/2/3/2/3 | | (5) ¼ + (3) ½  2/2∙2/2/2∙2/2/2∙2/2 | |
| 21 | **S**  **A** | (5) ½  2 odd/3 even | (5) ¼ + (3) ½  2/2∙2/2/2∙2/2/2∙2/2 | | (5) ½ + (3) ¼  2∙2/2/2∙2/2/2∙2/2/2∙2 | | (5) ¼ + (3) ¾  3/2∙2/3/2∙2/3/2∙2/3 | |
| 24.5 | **S**  **A** | (2) 1½  2 odd/2∙2 even | (5) ½ + (3) ¼  2∙2/2/2∙2/2/2∙2/2/2∙2 | | (5) ¾  3/5/3/5/3/5/3 | | (3) ½ + (5) ½  3 odd/5 even | |
| 28 | **S**  **A** | (2) ½ + (5) ½  3 odd/2∙2 even | (5) ¾  3/5/3/5/3/5/3 | | (3) 1 + (5) ¼  3/3∙3/3/3∙3/3/3∙2/3 | | (3) 1½  5 odd/2∙2 even | |
| 31.5 | **S**  **A** | (3) ½ + (5) ½  3 odd/5 even | (3) 1 + (5) ¼  3/3∙3/3/3∙3/3/3∙3/3 | | (2) ½ + (5) ¾  2∙2/3∙3/2∙2/3∙3/2∙2/3∙3/2∙2 | | (5) ¾ + (3) ½  3∙3/2∙2/3∙3/2∙2/3∙3/2∙2/3∙3 | |

Group S: (tablet strength; mg) tablet quantity per day

Group A: tablet strength∙tablet strength

**Supplementary Table S1.** Warfarin dosage adjustment protocol.
